# Supplementary material for: Early-life gut microbiome associates with positive vaccine take and shedding in neonatal schedule of the human neonatal rotavirus vaccine RV3-BB
Source: Nat Commun. 2025 Apr 11;16:3432. doi: 10.1038/s41467-025-58632-6 (PMC11986061; doi:10.1038/s41467-025-58632-6)
Supplement: Supplementary file 1 — Supplementary Information [file 41467_2025_58632_MOESM1_ESM.pdf]

Early-life gut microbiome is associates with positive vaccine take and shedding in neonatal schedule of the human neonatal rotavirus vaccine, RV3-BB

Josef Wagner[1,2,3]*, Amanda Handley[1,4], Celeste M. Donato[1,3], Eleanor A. Lyons[1], Daniel Pavlic[1], Darren Suryawijaya Ong[1], Rhian Bonnici[1], Nada Bogdanovic-Sakran[1], Edward P.K. Parker[5], Christina Bronowski[6], Jarir At Thobari[7,8], Cahya Dewi Satria[8], Desiree Witte[6,10], Hera Nirwati[9], Khuzwayo C. Jere[6,10], Ashley Mpakiza[10,11], Emma Watts[1], Ann Turner[10], Karen Boniface[1], Jonathan Mandolo[10], Frances Justice[1], Naor Bar-Zeev[6], Miren Iturriza-Gomara [6,12], Jim P. Buttery[1,3,13], Nigel A. Cunliffe[6], Yati Soenarto[8], Julie E. Bines[1,3,14]*

Legend of Supplementary Tables

Supplementary Table 1: Comparison in participant characteristics between in the per protocol population of the RV3-BB clinical vaccine trial and the RV3-BB Microbiome study cohort

Supplementary Table 2: Number of bacterial 16S RNA V3-V4 reads at the different stages through the MOTHUR and Oligotyping "MED" analysis pipeline

Indonesia study cohorts and the Rotarix Malawi and India study cohorts

Supplementary Table 3: Alpha and Beta diversity at key study timepoints in association with IgA seroconversion

Supplementary Table 4: Alpha and Beta diversity in association with vaccine response across all time points and vaccine doses in the RV3-BB Malawi and Indonesia study

Supplementary Table 5: Alpha and Beta diversity Cross-Analysis between the RV3-BB Indonesia Neonatal group and the Placebo group

Supplementary Table 6: PERMANOVA Beta-diversity analysis for the Malawi RV3-BB dataset

Supplementary Table 7: PERMANOVA Beta-diversity analysis for the Indonesia RV3-BB dataset

associations between microbial taxa and vaccine variables in the Malawi and Indonesia RV3-BB cohorts

Supplementary Table 8: Participant characteristics in the Rotarix Malawi and India study cohorts

Supplementary Table 9: Multivariable statistical framework analysis (MaAsLin2) for finding associations between microbial taxa and vaccine variables in the Rotarix Indian and Malawi study cohorts

Legend of Supplementary Figures

Supplementary Fig. 1 Alpha diversity analysis for the Rv3-BB Indonesia study cohort in participants with positive or negative vaccine response in the Neonatal vaccine schedule group

The alpha diversity was analyzed between the negative vaccine response group (N) and the positive vaccine response group (Y) for the vaccine variables "Vaccine take" and “Shedding" at week 1 shown in **a**, at week 14 shown in **b**, and at week 18 shown in **c**. The analysis was conducted on three vaccine doses: dose 1 (d1), dose 3 (d3), and dose 4 (marked as d4*as this time point is after three doses of vaccine and one dose of placebo). The data were tested for normal distribution using the Shapiro-Wilk and Kolmogorov-Smirnov tests. For normally distributed data, a two-tailed unpaired t-test was employed, whereas for non-normally distributed data, a two-tailed unpaired Mann-Whitney test was used. Data are presented in a box and whisker plot. The box extends from the 25th to the 75th percentile and the line in the middle is plotted at the median. The whiskers represent the 10-90 percentiles. All data points outside the 10-90 percentile are shown. All the individual numbers used for box plot generation are presented in Supplementary Table 4. All statistical tests were conducted in GraphPad Prism 10 for macOS (v 10.3.0).

Supplementary Fig. 2 PCoA showing that the microbiome in the RV3-BB Malawi infant and neonatal schedule groups is not different

The PCoA based on the Bray-Curtis distance matrix performed between the infant and neonatal groups in the Malawi RV3-BB vaccine cohort showed that the microbiome was not different between these two groups at **a** baseline, **b** week1, **c** week6, and **d** week 10. The PERMANOVA test for beta diversity confirmed that the microbiome was not statistically different between the infant and neonatal groups.

Supplementary Figure 3: PCoA for gender and antibiotic use in the RV3-BB Malawi and Indonesia study cohorts

PCoA based on Bray-Curtis distance matrix between gender and antibiotics free group and the group who has received antibiotics during the entire 18 weeks IP dose period did not reveal different bacterial clusters between the different groups and timepoints analyzed. The explained variance for the coordinates are shown in brackets next to the coordinate axis label. PCoA was done with the Paleontological Statistic software package for education and data analysis (v PAST 4.04) with 9999 permutations on total sum scaling (TSS) transformed data. The confidence ellipse was drawn at 60%.

Supplementary Figure 4: Principal Component Analysis (PCA) for timepoints in the RV3-BB Malawi and Indonesia study cohorts demonstrate the existence of age-related distinct clusters

a PCA conducted on the Malawi study all samples combined (n=355), **b** using neonatal vaccine schedule group samples (n=272), **c** and infant vaccine schedule group samples (n=83) shows the presence of two age-related distinctive bacterial clusters (at baseline and week 1 samples; and at week 6 and week 14). This distinct age-related bacterial cluster was also observed in the Indonesia study cohort; **d** in the combined (neonatal vaccine schedule group and infant vaccine schedule group; n= 478), **e** in the neonatal vaccine schedule group at week 1 compared to the week 14 and 18 cluster, **f** and in the infant vaccine schedule group the week 1 compare to week 18. **g** In the placebo group this age-related bacterial cluster was also observed between the week 1 and week 14 and 18 (g). The PCA plot was overlayed with a biplot showing the most distinctive bacterial cluster responsible for the separation.

Supplementary Table 1

Comparison in participant characteristics between in the per protocol population of the RV3-BB clinical vaccine trial and the RV3-BB Microbiome study cohort

Variables	RV3-BB Malawi Study		RV3-BB Indonesia Study	
	**Per Protocol Population**	**Microbiome Sub study**	**Per Protocol Population**	**Microbiome Sub study**
Number of participants<br>Number of microbiome samples	565	186<br>355	1513	193<br>478
Gender Female: n (%)	270 (47.8%)	90 (48.4%)	722 (47.7%)	92 (47.7%)
Gestational age:<br>Number of values<br>Mean weeks (SD)	561<br>37.5 (1.13)	184<br>37.5 (1.121)	1512<br>39.54 (1.1)	193<br>39.53 (1.052)
Birth weight:<br>Number of values<br>Mean grams (SD)	563<br>3119.9 (362.83)	186<br>3069 (338.5)	1513<br>3119.94 (339.85)	193<br>3091 (339.85)
Age at first dose of IP:<br>Number of values<br>Mean days (SD)	565<br>1.5 (1.43)	186<br>1.34 (1.359)	1513<br>3.5 (1.3)	192<br>3.141 (3)

Supplementary Table 2: Number of Bacterial 16S RNA V3-V4 reads at the different stages trough the MOTHUR and Oligotyping "MED" analysis pipeline

			RV3-BB Indonesia and Malawi microbiome study		Rotarix India and Malawi microbiome study*	
Program	read number through the pipeline		Indonesia	Malawi	India	Malawi
MOTHUR v1.45.2 + v.1.47.1	total number or raw reads (# samples)		55,081,070 (483)	65,407,416 (373)	196,757,612 (1147)	50,7455,38 (283)
	total number of quality screen reads		40,346,048	48,312,246	162,244,783	44,766,568
	generate non redundant reads	# of unique seqs:	9,250,041	10,468,085	6,594,916	21,002,943
	good aligned	# of unique seqs:	8,870,216	9,768,850	20,588,097	6,470,646
use Silva.nr -v138_1.align		total # of seqs:	39,619,128	46,771,608	161,528,066	44,567,637
	preclustered	# of unique seqs:	4,524,995	4,528,581	11,664,277	2,947,110
		total # of seqs:	39,619,128	46,771,608	161,528,066	44,567,637
vsearch v2.16.0_macos_x86_64	chimera free	# of unique seqs:	3,578,213	3,581,137	9,771,385	2,745,306
use for chimera removal		total # of seqs:	37,026,965	43,146,431	151,984,063	43,842,314
use Silva.nr_v138_1.align	correct classified	# of unique seqs:	3,574,969	3,577,310	9,770,721	2,745,118
use Silva.nr_v138_1.tax	(Final high quality reads in % from raw reads)	total # of seqs:	37,023,703 (67.22%)	43,140,063 (65.96%)	151,982,642 (77.2%)	43,842,032 (86.4%)
	subsampled size		40,272<br>5 negative controls had btw 383-1056 reads. 11 samples had betw 28502-39676 reads. All 16 samples were added back in to the data set	65,745<br>One negative control with 778 reads and one sample with 13606 reads were added back in to the data set	50,000<br>3 samples had btw 25,598 and 44,077 reads and were added back into the dataset.	50,000<br>29 samples had btw 25,474 and 49,989 reads and were added back into the dataset
Non-redundant (MOTHUR "deunique and renamed" reads used for MED oligotyping		total # of seqs:	**19,210,681**	**24,412,719**	**57,048,980**	**14,098,237**

		RV3-BB Indonesia and Malawi microbiome study		Rotarix India and Malawi microbiome study*	
		Indonesia	Malawi	India	Malawi
**Oligotyping pipeline**		MED oligotyping	MED oligotyping	MED oligotyping	MED oligotyping
Log data from:	**Number of sequences analyzed**	**19,210,681**	**24,412,719**	**57,048,980**	**14,098,237**
Minimum Entropy Decomposition	Number of samples found (including controls)	483 (478 are microbiome samples**)	373 (355 are microbiome samples**)	1147 (#2)	283
using Oligotyping -M 100 -V 2	Number of characters in each alignment	1786	1909	1939	1781
	Average read length (without gaps)	465	445	446	446
	» Handling Outliers				
	Final number of outliers due to -M	1,663,344	1,578,696	1,883,317	603,118
	Final number of outliers due to -V	1,151,327	1,176,493	2,237,414	461,387
	Final total number of outliers	2,814,671	2,755,189	4,120,731	1,064,505
	» Nodes				
	Number of sequences analyzed	19,210,681	24,412,719	57,048,980	14,098,237
The number of final nodes will be	Number of sequences represented after quality filtering	16,396,010 (85.35%)	21,657,530 (88.71%)	52,928,249	13,033,732
used for taxonomic classification	Number of raw nodes (before the refinement)	5,338	4,282	5,592	2,818
and detailed analysis	**Number of final nodes (after the refinement)**	**5,563**	**4,600**	**6,370**	**2,922**

RV3-BB microbiome study in Indonesia and Malawi (blue columns). Rotarix Study in India and Malawi (orange columns)

Rotarix microbiome study dataset includes bacterial 16S V3V4 sequence reads downloaded from the European Nucleotide Archive (Accession code PRJEB38948). Only samples used in the final analysis by Parker et al were included in this analysis .

*NATURE COMMUNICATIONS | (2021)12:7288 | https://doi.org/10.1038/s41467-021-27074-1 | www.nature.com/naturecommunications

# = number

#2 = two samples were removed following the finishing of the oligotyping pipeline because they were miss-classified and a part of the Malawi cohort.

Supplementary Table 3: Alpha and Beta diversity at key study timepoints in association with IgA seroconversion

Dose 4* = IP dose 4 in the neonatal vaccine schedule which is 3 doses of vaccine followed by one dose of placebo (Figure 1) Dose 4* = IP dose 4 in the infant vaccine schedule group which is one dose of placebo followed by 3 doses of vaccine. NA = sample not available for analysis. Alpha diversity indexes for Fisher's alpha index, Simpson index and Richness measure were compared with positive("yes") and negative ("no") IgA seroconversion groups across treatment allocation groups. Data were checked for normality using the Anderson-Darling test and the Shapiro-Wilk test. If all the "yes" and "no" data within each variable dose group were normally distributed with both tests, then the parametric two-tailed t test was used, otherwise the non-parametric two-tailed Mann-Whitney test was used. The analysis was conducted in GraphPad Prism 9 for macOS.

	Malawi RV3-BB Study				
	Neonatal Vaccine Schedule Group			Infant Vaccine Schedule Group	
	**Dose 1** Vaccine	**Dose 2** Vaccine	**Dose 4 *** Placebo	**Dose 1** Placebo	**Dose 4^** Vaccine
**BASELINE**					
Number of participants with positive IgA seroconversion/total	9/41	15/41	20/41	0/18	8/18
Alpha diversity:					
Fishers alpha index	0.5455	0.1652	0.0655	NA	0.9454
Simpson's index	0.7925	0.718	0.4935	NA	0.1935
Observed richness measure	0.7286	0.3995	0.128	NA	0.4009
Beta diversity:					
R2 value	0.028	0.012	0.021	NA	0.024
P value	0.3286	0.8545	0.5174	NA	0.8621
**WEEK 1**					
Number of participants with positive IgA seroconversion/total	7/36	12/36	19/36	0/11	6/11
Alpha diversity:					
Fishers alpha index	0.5479	0.7624	0.8802	NA	0.9307
Simpson's index	0.5836	0.0945	0.3969	NA	0.3313
Observed richness measure	0.9711	0.4531	0.6724	NA	0.7629
Beta diversity:					
R2 value	0.019	0.012	0.013	NA	0.114
P value	0.7084	0.8928	0.8612	NA	0.336
**WEEK 6**					
Number of participants with positive IgA seroconversion/total	19/103	29/103	59/103	0/27	13/27
Alpha diversity:					
Fishers alpha index	0.3128	0.3164	0.8848	NA	0.6678
Simpson's index	0.8166	0.8925	0.7881	NA	0.1252
Observed richness measure	0.3135	0.3963	0.6784	NA	0.807
Beta diversity:					
R2 value	0.006	0.007	0.013	NA	0.061
P value	0.7814	0.6435	0.2077	NA	0.1373
**WEEK 14**					
Number of participants with positive IgA seroconversion/total	16/92	29/63	48/92	0/27	13/27
Alpha diversity:					
Fishers alpha index	0.1674	0.7715	0.268	NA	0.8408
Simpson's index	0.3285	0.3192	0.3852	NA	0.1632
Observed richness measure	0.2168	0.9135	0.4379	NA	0.43
Beta diversity:					
R2 value	0.009	0.011	0.013	NA	0.04`
P value	0.5605	0.4231	0.3152	NA	0.3518

	Indonesia RV3-BB Study		
	**Neonatal Vaccine Schedule Group**	**Infant Vaccine Schedule Group**	**Placebo Group**
	**Dose 1** Vaccine		**Dose 1** Placebo
**WEEK 1**			
Number of participants with positive IgA seroconversion/total	13/62		13/62
Alpha diversity:			
Fishers alpha index	0.6988	NA	0.8913
Simpson's index	0.6988	NA	0.3547
Observed richness measure	0.7451	NA	0.7977
Beta diversity:			
R2 value	0.013	NA	0.015
P value	0.6261	NA	0.4901
	**Dose 3** Vaccine		**Dose 3** Placebo
**WEEK 14**			
Number of participants with positive IgA seroconversion/total	44/60		24/54
Alpha diversity:			
Fishers alpha index	0.9756	NA	0.9656
Simpson's index	0.5677	NA	0.7494
Observed richness measure	0.9913	NA	0.7895
Beta diversity:			
R2 value	0.015	NA	0.021
P value	0.4901	NA	0.3115
	**Dose 4 *** Placebo	**Dose 4^** Vaccine	**Dose 4** Placebo
**WEEK 18**			
Number of participants with positive IgA seroconversion/total	42/53	54/63	34/56
Alpha diversity:			
Fishers alpha index	0.5958	0.5984	0.5107
Simpson's index	0.7375	0.4678	0.3461
Observed richness measure	0.5193	0.5524	0.6598
Beta diversity:			
R2 value	0.017	0.01	0.01
P value	0.4646	0.6887	0.8046

Malawi - Alpha and Beta diversity	Neonatal group						Infant group				Placebo group					
	Vaccine take			Stool shedding			Vaccine take		Stool shedding		Vaccine take			Stool shedding		
	Dose 1	Dose 2	Dose 4	Dose 1	Dose 2	Dose 4	Dose 1	Dose 4	Dose 1	Dose 4	Dose 1	Dose 3	Dose 4	Dose 1	Dose 3	Dose 4
Treatment allocation	Vaccine	Vaccine	Placebo	Vaccine	Vaccine	Placebo	Placebo	Vaccine	Placebo	Vaccine	Placebo	Placebo	Placebo	Placebo	Placebo	Placebo
BASELINE																
Participants with positive vaccine take or stool shedding/total participants	9/41	24/41	31/41	2/41	16/41	22/41	8/18	15/18	7/18	15/18	No placebo group					
Alpha diversity:																
Fishers alpha analysis	0.5299	**0.0373**	**0.0137**	0.7537	0.3501	0.1614	0.4598	0.9118	0.8601	0.9118						
Simpson index	0.7273	0.7782	0.5242	0.7549	0.7559	0.5467	0.2531	0.6063	0.3529	0.6063						
Richness measure	0.8649	0.0696	**0.022**	0.7817	0.2284	0.1529	0.706	0.9118	0.9976	0.9118						
Beta diversity:																
R2 value	0.028	0.016	0.023	0.045	0.042	0.035	0.058	0.035	0.03	0.035						
P value	0.3228	0.716	0.468	0.0892	0.1094	0.1947	0.4106	0.7245	0.777	0.7249						
WEEK 1																
Participants with positive vaccine take or stool shedding/total participants	12/36	23/36	31/36	7/36	19/36	25/36	6/11	5/11	10/11	8/11	No placebo group					
Alpha diversity																
Fishers alpha analysis	0.0866	0.0644	0.2401	**0.0232**	0.0862	0.4154	0.2795	0.538	0.4087	0.35						
Simpson index	0.0669	**0.0337**	**0.0215**	0.0537	**0.0275**	0.0927	0.3152	0.3606	0.3643	0.5022						
Richness measure	**0.0124**	**0.0151**	0.1138	**0.0011**	**0.0123**	0.2172	0.2767	0.4131	0.2964	0.2192						
Beta diversity:																
R2 value	0.063	0.071	0.04	0.101	0.056	0.048	0.04	0.047	0.015	0.044						
P value	**0.0397**	**0.0192**	0.1969	**0.0014**	0.0629	0.111	0.8399	0.9115	0.9699	0.8104						
WEEK 6																
Participants with positive vaccine take or stool shedding/total participants	27/103	71/103	89/103	14/103	57/103	67/103	16/27	21/27	13/27	20/27	No placebo group					
Alpha diversity:																
Fishers alpha analysis	0.6552	**0.0039**	**0.0115**	0.2392	**0.0013**	**0.0002**	0.7283	0.5609	0.81	0.8868						
Simpson index	0.8059	0.6042	0.2988	0.5072	0.8511	0.8197	0.909	0.4852	0.845	0.8165						
Richness measure	0.5644	**0.0002**	**0.0016**	0.3661	**<0.0001**	**<0.0001**	0.7526	0.7134	0.4295	0.63						
Beta diversity:																
R2 value	0.011	0.03	0.025	0.018	0.046	0.041	0.048	0.037	0.44	0.027						
P value	0.3067	**0.0055**	**0.0195**	0.079	**0.0003**	**0.0003**	0.2571	0.4293	0.3064	0.6446						
WEEK 14																
Participants with positive vaccine take or stool shedding/total participants	25/92	55/92	73/92	14/92	41/92	52/92	14/27	25/27	10/27	21/27	No placebo group					
Alpha diversity:																
Fishers alpha analysis	**0.0141**	0.1013	0.9923	**0.0462**	**0.0107**	0.0608	0.8685	0.9249	0.4546	0.3496						
Simpson index	0.5341	0.5669	0.2523	0.6966	0.8191	0.2556	0.4036	0.6606	0.4347	0.8696						
Richness measure	**0.0257**	0.1926	0.9786	0.0912	0.1008	0.2218	0.5656	0.9061	0.5152	0.1606						
Beta diversity:																
R2 value	0.012	0.021	0.025	0.006	0.015	0.012	0.024	0.0245	0.011	0.135						
P value	0.3304	0.067	**0.037**	0.8327	0.2042	0.349	0.7053	0.6235	0.9444	**0.0082**						

Indonesia - Alpha and Beta diversity	Neonatal group						Infant group				Placebo group					
	Vaccine take			Stool shedding			Vaccine take		Stool shedding		Vaccine take			Stool shedding		
	Dose 1	Dose 3	Dose 4	Dose 1	Dose 3	Dose 4	Dose 1	Dose 4	Dose 1	Dose 4	Dose 1	Dose 3	Dose 4	Dose 1	Dose 3	Dose 4
Treatment allocation	Vaccine	Vaccine	Placebo	Vaccine	Vaccine	Placebo	Placebo	Vaccine	Placebo	Vaccine	Placebo	Placebo	Placebo	Placebo	Placebo	Placebo
WEEK 1																
Participants with positive vaccine take or stool shedding/total participants	14/59	56/59	57/59	2/59	39/59	39/59	1/64	64/64	1/64	15/64	13/64	27/64	39/64	2/63	4/64	4/64
Alpha diversity:																
Fishers alpha analysis	0.4116	0.0584	0.3179	0.3179	0.153	0.3277	NA	NA	NA	0.53	0.5413	0.4739	0.7638	>0.999	0.8201	0.8201
Simpson index	0.8533	0.9096	0.9492	0.1286	0.4695	0.5728	NA	NA	NA	0.068	0.7291	0.2334	0.6131	0.7465	0.2083	0.216
Richness measure	0.5197	0.0829	0.3998	0.422	0.852	0.2107	NA	NA	NA	0.6379	0.9343	0.1545	0.913	0.8315	0.5288	0.5288
Beta diversity:																
R2 value	0.011	0.023	0.038	0.0173	0.054	0.0122	0.016	0.013	0.016	0.040	0.016	0.0215	0.0105	0.046	0.046	0.0127
P value	0.7153	0.2083	**0.043**	0.4141	0.0991	0.6486	0.4168	0.5803	0.4195	0.6895	0.4192	0.2136	0.7002	0.109	0.0997	0.5814
WEEK 14																
Participants with positive vaccine take or stool shedding/total participants	14/58	57/60	58/60	2/58	41/60	41/60	No microbiome analysis		No microbiome analysis		12/56	25/56	34/56	2/55	4/56	4/56
Alpha diversity:																
Fishers alpha analysis	0.4772	0.3591	0.4746	0.2202	**0.0008**	**0.0010**					0.6405	0.8190	0.6953	0.9455	0.6385	0.4506
Simpson index	0.7669	0.3958	0.4079	0.4598	0.2172	0.2356					0.4958	0.7561	0.5771	0.6815	0.2703	0.2703
Richness measure	0.4527	0.3247	0.4520	0.1887	**0.0006**	**0.0005**					0.5605	0.7685	0.7337	0.9455	0.4695	0.4695
Beta diversity:																
R2 value	0.024	0.024	0.030	0.022	0.103	0.110					0.004	0.009	0.006	0.017	0.022	0.022
P value	0.7218	0.1896	0.106	0.7714	**0.0001**	**0.0001**					0.9785	0.841	0.9469	0.4701	0.2837	0.2777
WEEK 18																
Participants with positive vaccine take or stool shedding/total participants	13/52	51/54	52/54	2/52	35/54	35/54	1/64	64/64	1/64	15/64	10/58	24/58	35/58	11/56	2/58	2/58
Alpha diversity:																
Fishers alpha analysis	0.3044	0.4248	0.5590	0.3394	0.2901	0.2918	NA	NA	NA	0.0525	0.5360	0.3992	0.4033	NA	0.6400	0.6400
Simpson index	0.8022	0.3824	0.9811	0.6335	0.5295	0.5901	NA	NA	NA	0.3873	0.1429	0.2416	0.2753	NA	0.2904	0.2904
Richness measure	0.6410	0.3237	0.5311	0.4615	0.2409	0.2352	NA	NA	NA	0.0691	0.2176	0.2612	0.3512	NA	0.5336	0.5336
Beta diversity:																
R2 value	0.022	0.048	0.037	0.039	0.058	0.031	0.015	0.005	0.015	0.036	0.012	0.018	0.012	0.010	0.006	0.006
P value	0.853	**0.0313**	0.0916	0.4071	0.1066	0.1183	0.4046	0.9328	0.4057	0.6746	0.6843	0.3821	0.7083	0.7888	0.9471	0.4385

Supplementary Table 4: Alpha and Beta diversity in association with vaccine response across all time points and vaccine doses in the RV3-BB Malawi and Indonesia study

Differences in alpha and beta diversity between participants who were positive for vaccine take and stool shedding and those who were negative were determined for three alpha diversity indices (Fishers alpha, Simpson index and Richness measure) and one beta diversity measure (permuted multivariate analysis of variance (PERMANOVA) test with Bray-Curtis measure). For beta diversity, R^2 and p values are reported. For alpha diversity, the p-value is given. Significant p-values with 95% confidence intervals are shown in bold. All significant and non-significant p-values are reported with 4 decimals where applicable. NA = no statistical tests were performed for the following reasons: either no data were available for one of the groups or there were not at least two values for one of the groups. Data were tested for Gaussian distribution using the Shapiro-Wilk and Kolmogorov-Smirnov tests. For normally distributed data, the two-tailed unpaired parametric t-test was used for statistical analysis. For non-normally distributed data, the two-tailed unpaired non-parametric Mann-Whitney test was used. P values were not corrected for multiple time points. Benjamini-Hochberg corrected p values at key study timepoints are shown in Table 2.

Supplementary Table 5: Alpha and Beta divesity Cross-Analysis between the RV3-BB Indonesia Neonatal group and the Placebo group

	Stool shedding			
	Neonatal Schedule group	Placebo group	Neonatal Schedule group	Placebo group
	Vaccine dose3	Placebo dose3	Vaccine/Placebo dose 4*	Placebo dose 4
**WEEK 14**				
**Participants: total number**	60	60	60	56
Participants in neonatal schedule group with positive shedding: number (%)	41 (68%)		41 (68%)	
Alpha diversity:				
Fishers alpha analysis	**0.0027**		**0.0025**	
Richness measure	**0.0016**		**0.0015**	
Beta diversity:				
R2 value	0.014		0.01484	
P value	0.1992		0.1788	
**WEEK 18**				
**Participants: total number**	54	48	54	58
Participants in neonatal schedule group with positive shedding: number (%)	35 (64.8%)		35 (64.8%)	
Alpha diversity:				
Fishers alpha analysis	0.0627		0.0525	
Richness measure	0.0629		0.0517	
Beta diversity:				
R2 value	0.008		0.008	
P value	0.6585		0.6657	

Bold value indicates statistically significant comparions

dose 4* denotes IP dose 4 in the neonatal schedule which is following three doses of vaccine and one dose of placebo

Supplementary Table 6. PERMANOVA Beta-diversity analysis for the Malawi RV3-BB dataset

MALAWI (n = 355)	N	INFANT	N	NEONATAL	PERMANOVA across all samples within a time point, testing for group differences
Baseline	18	gastro_18wks R^2=0.17, F=3.24, P=0.0307	41	MofD R^2=0.13, F=5.8, P=0.0004	group differences between infant and neonatal at baseline analysed together with other variables # P = 0.5363
IP dose 1 (week1)	11	no significant tests	36	MofD R^2=0.11, F=4.28, P=0.0003<br>birth weight R^2=0.06, F=2.44, P=0.0248	group differences between infant and neonatal at baseline analysed together with other variables # P = 0.1235
IP dose 2 (week 6)	27	no significant tests	103	no significant tests	group differences between infant and neonatal at baseline analysed together with other variables # P = 0.5368
IP dose 4 (week 14)	27	no significant tests	92	no significant tests	group differences between infant and neonatal at baseline analysed together with other variables ## P = 0.5523
PERMANOVA for timepoints	83	Timepoints in the infant group analysed together with variables **<br>R^2=0.28, F=10.6, P=0.0001	272	Timepoints in the neonatal group analysed together with variables **<br>R^2=0.22, F=26.2, P=0.0001	The inclusion of other additonal variables was done to check for confounding effects of the additional added variables. None of them showed a confounded effect.

The one-sided PERmutational **M**ultivariate **AN**alysis **O**f **VA**riance (PERMANOVA) was performed using the "adonis2" function from the
PERMANOVA test was performed
(1) across all samples within a study group, testing for timepoint differences (results in orange highlighted section)
(2) across all samples within a timepoint, testing for group differences (results in blue highlighted section)
(3) across an individual timepoint and study group (results in green highlighted section)
All statistically significant tests are presented in red font, reporting the R2 value, the F value and the P value
We added all patients and clinical variables available to each PERMANOVA test, to check for any confoudning effects

variables *	adonis2 (Y ~ gastro_18wks+birth_weight+abx_18wks+MofD+breastfeeding_18wks+timepoints, permutations = 9999, data = meta, method = "bray")
variables **	adonis2 (Y ~ gastro_18wks+birth_weight+abx_18wks+MofD+timepoints, permutations = 9999, data = meta, method = "bray")
variables #	adonis2 (Y ~ gastro_18wks+birth_weight+abx_18wks+MofD+group, permutations = 9999, data = meta, method = "bray")
variables ##	adonis2 (Y ~ gastro_18wks+birth_weight+abx_18wks+MofD+breastfeeding_18wks+group, permutations = 9999, data = meta, method = "bray")
Baseline (Infant)	adonis2 (Y ~ birth_weight+abx_18wks+MofD+gastro_18wks, permutations = 9999, data = meta, method = "bray")
Baseline (Neonatal)	adonis2 (Y ~ gastro_18wks+birth_weight+abx_18wks+MofD, permutations = 9999, data = meta, method = "bray")
Week 1 (Neonatal)	adonis2 (Y ~ gastro_18wks+birth_weight+abx_18wks+MofD, permutations = 9999, data = meta, method = "bray")
Week 1 (Neonatal)	adonis2 (Y ~ gastro_18wks+abx_18wks+MofD+birth_weight, permutations = 9999, data = meta, method = "bray")

MofD = Mode of Delivery
Pairwise PERMANOVA done in PAST 4 between baseline, week 1, week 6 and week 14 timepoints

		INFANT		NEONATAL
Bonferroni corrected P value between		baseline and week 1 = 0.2202<br>baseline and week 6 = 0.0006<br>baseline and week 14 = 0.0006<br>week 1 and week 6 = 0.003<br>week 1 and week 14 = 0.0012<br>week 6 and week 14 = 1		baseline and week 1 = 0.1<br>baseline and week 6 = 0.0006<br>baseline and week 14 = 0.0006<br>week 1 and week 6 = 0.0006<br>week 1 and week 14 = 0.0006<br>week 6 and week 14 = 0.5658
F value between		baseline and week 1 = 2.448<br>baseline and week 6 = 17.93<br>baseline and week 14 = 22.2<br>week 1 and week 6 = 6.992<br>week 1 and week 14 = 10.89<br>week 6 and week 14 = 1.105		baseline and week 1 = 1.099<br>baseline and week 6 = 36.86<br>baseline and week 14 = 48.23<br>week 1 and week 6 = 29.81<br>week 1 and week 14 = 40.85<br>week 6 and week 14 = 1.787

PAST = Paleontological Statistics software package for education and data analysis (version 4.10)

Supplementary Table 7. PERMANOVA Beta-diversity analysis for the Indonesia RV3-BB dataset

**IP dose** | timepoint (number)	N	PLACEBO	N	NEONATAL	N	INFANT	PERMANOVA with study groups (placebo, neonatal, and infant) + variables **
**IP dose 1 |** Day 1 to 5 (n = 187)	64	breastfeeding R2=0.03, F=2.2, P=0.0421 #	59	birth weight R2=0.05, F= 3.23, P=0.0046 #<br>MofD R2=0.04, F=2.42, P=0.0311 #	64	no significant tests	groups + variables no significant values
**IP dose 3 |** Week 14 to 16 (n = 116)	56	no significant tests	60	birth weight R2=0.04, F= 2.67, P=0.0203 #	0	no data	groups + variables no significant values
**IP dose 4 |** Week 18 to 20 (n = 175)	57	breastfeeding R2=0.05, F=2.77, P=0.0161 #	54	no significant tests	64	no significant tests	groups + variables no significant values
PERMANOVA for IP dose + variables *	177	R2=0.14, F=28.1, P=0.0001	173	R2=0.14, F=28.46, P=0.0001	128	R2=0.16, F=24.01, P=0.0001	

The one-sided PERmutational **M**ultivariate **AN**alysis **O**f **VA**riance (PERMANOVA) was performed using the "adonis2" function from the VEGAN package in R.

PERMANOVA test was performed

(1) across all samples within a study group, testing for IP dose (timepoint differences) (results in orange highlighted section)

(2) across all samples within a IP dose (timepoint), testing for group differences (results in blue highlighted section)

(3) across an individual IP dose (timepoint) and study group (results in green highlighted section)

All statistically significant tests are presented in red font, reporting the R2 value, the F value and the P value

We added all patients and clinical variables available to each PERMANOVA test, to check for any confoudning effects

MofD = Mode of Delivery

variables * feeding_upto5mths+V_Ipdose, permutations = 9999, data = meta, method = "bray")

variables ** ıstfeeding_upto5mths+group, permutations = 9999, data = meta, method = "bray")

default # + breastfeeding_upto5mths, permutations = 9999, data = meta, method = "bray")

if any of the default order of variables was signficant, that variable was then placed at the last positon in the adonis2 function from which the R2, F, and P value are shown

Pairwise PERMANOVA done in PAST 4 between IP dose 1, 3, and 4

between IP dose 1, 3, and 4		PLACEBO		NEONATAL
Bonferroni corrected P value between		IP dose 1 and 3 = 0.003<br>IP dose 1 and 4 = 0.003<br>IP dose 3 and 4 = 1		IP dose 1 and 3 = 0.003<br>IP dose 1 and 4 = 0.003<br>IP dose 3 and 4 = 1
F value between		IP dose 1 and 3 = 16.92<br>IP dose 1 and 4 = 22.32<br>IP dose 3 and 4 = 0.6981		IP dose 1 and 3 = 18.68<br>IP dose 1 and 4 = 22.28<br>IP dose 3 and 4 = 0.781

PAST = Paleontological Statistics software package for education and data analysis (version 4.10)

Supplementary Table 8. Description of Rotarix participants in Malawi and India microbiome studies

	Malawi	India
Number of participants	107	307
Number of samples	283	1145
Gender: number female (%)	55 (51.4%)	152 (49.5%)
Exclusive breastfeeding to 11 weeks: number (%)	96 (89.7%)	256 (83.4%)
Exposed to antibiotics birth to week 14: number (%)	28 (26.2%)	84 (27.4%)
HIV exposure: number of participants with known HIV exposure status	23	0
Rotavirus exposure in week 1*: number	9 (22 unknown)	166 (3 unknown)
Administered Inactivated poliovirus vaccine: number	0	100
Age at first dose of vaccine: mean days (standard deviation)	42.01 (0.6707)	42.6 (.343)
Birth weight: mean in kg (standard deviation)	3.013 (0.4249)	2.957 (0.4228)
Weight at 6 to 8 weeks of age: number of participants with data	101	306
Weight at 6 to 8 weeks of age: mean in kg (standard deviation)	4.506 (0.5906)	4.204 (0.553)

**Number of samples for microbiome analysis**	**Malawi**	**India**
Total number	283	1145
Study Week 1	81	289
Study Week 4	75	287
Study Week 6 = post dose 1 shedding	66	287
Study Week 10 = post- dose 2 shedding	61	282

*Pre-vaccination RV exposure defined as detection of rotavirus shedding in week of life 1 (VP6 Ct<35) or baseline seropositivity (BB1_IgA>20)

All of the individuals in the "final analysis set" (108 babies from Malawi and 307 babies from India) received 2 doses of Rotarix.

Supplementary Table 9. Multivariable statistical framework analysis (MaAsLin2) for finding associations between microbial taxa and vaccine variables in the India and Malawi Rotarix cohorts

**INDIA time**	**analysis group**	**method**	**value for shedding**	**feature**	**min 0.1% abundance in the total cohort**	**metadata**	**value**	**coef**	**stderr**	**N**	**min 5 N.not.0**	**pval**	**qval**	**min 10% sample with taxa preval. in a given analysed dataset**
week1	dose 2 shedding	univariate	NSP2+VP6	*Lachnospiraceae*	0.71	dose2_shedding_NSP2VP6	yes	2.219	0.912	288	67	0.01562	0.14843	23.26
week1	dose 2 shedding	univariate	NSP2+VP6	*Prevotella_9*	0.48	dose2_shedding_NSP2VP6	yes	0.960	0.403	288	38	0.01784	0.14888	13.19
week4	cumulative shedding	univariate	NSP2+VP6	*Escherichia Shigella*	12.98	cum_shedding_NSP2VP6	yes	8.358	2.343	284	275	0.00042	0.05498	96.83
week4	cumulative shedding	multivariate	NSP2+VP6	*Escherichia Shigella*	12.98	cum_shedding_NSP2VP6	yes	9.623	2.373	284	275	0.00007	0.01539	96.83
week4	dose 2 shedding	univariate	NSP2+VP6	*Escherichia Shigella*	12.98	dose2_shedding_NSP2VP6	yes	10.745	3.126	286	277	0.00068	0.04393	96.85
week4	dose 2 shedding	multivariate	NSP2+VP6	*Escherichia Shigella*	12.98	dose2_shedding_NSP2VP6	yes	10.655	3.105	286	277	0.00069	0.11584	96.85
week4	dose 1 shedding	univariate	NSP2+VP6	*Lachnospiraceae*	0.71	dose1_shedding_NSP2VP6	yes	1.320	0.550	285	81	0.01704	0.20144	28.42
week6	dose 1 shedding	multivariate	NSP2+VP6	*Klebsiella*	4.76	dose1_shedding_NSP2VP6	yes	6.486	2.049	285	264	0.00173	0.16018	92.63
week6	dose 1 shedding	multivariate	NSP2+VP6	*Lacticaseibacillus*	0.11	dose1_shedding_NSP2VP6	yes	0.433	0.147	285	33	0.00342	0.21706	11.58
week6	dose 2 shedding	univariate	NSP2+VP6	*Clostridium_sensu_stricto*	0.51	dose2_shedding_NSP2VP6	yes	1.783	0.557	286	85	0.00151	0.10137	29.72
week6	dose 2 shedding	multivariate	NSP2+VP6	*Clostridium_sensu_stricto*	0.51	dose2_shedding_NSP2VP6	yes	1.682	0.562	286	85	0.00301	0.20175	29.72
week10	dose 2 shedding	univariate	NSP2+VP6	*Escherichia Shigella*	12.98	dose2_shedding_NSP2VP6	yes	7.239	2.488	281	276	0.00391	0.14395	98.22
week10	cumulative shedding	multivariate	NSP2	*Bifidobacterium*	43.98	cum_shedding_NSP2	yes	-8.758	2.417	279	277	0.00035	0.05099	99.28

**MALAWI time**	**analysis group**	**method**	**value for shedding**	**feature**	**min 0.1% abundance in the total cohort**	**metadata**	**value**	**coef**	**stderr**	**N**	**min 5 N.not.0**	**pval**	**qval**	**min 10% sample with taxa preval. in a given analysed dataset**
week4	dose 1 shedding	univariate	NSP2+VP6	*Collinsella*	2.31	dose1_shedding_NSP2VP6	yes	2.178	0.618	64	43	0.00081	0.10000	67.19
week4	dose 1 shedding	multivariate	NSP2+VP6	*Collinsella*	2.31	dose1_shedding_NSP2VP6	yes	2.403	0.705	64	43	0.00123	0.11846	67.19
week4	dose 1 shedding	multivariate	NSP2+VP6	*Ligilactobacillus*	0.38	dose1_shedding_NSP2VP6	yes	1.022	0.366	64	14	0.00717	0.22236	21.88
week4	dose 2 shedding	univariate	NSP2+VP6	*Prevotellaceae*	0.24	dose2_shedding_NSP2VP6	yes	1.734	0.583	52	6	0.00451	0.06491	11.54
week4	dose 2 shedding	univariate	NSP2+VP6	*Phascolarctobacterium*	0.13	dose2_shedding_NSP2VP6	yes	0.411	0.148	52	10	0.00767	0.08739	19.23
week4	dose 2 shedding	univariate	NSP2+VP6	*Succinivibrio*	0.77	dose2_shedding_NSP2VP6	yes	0.655	0.259	52	7	0.01471	0.11181	13.46
week6	dose 1 shedding	multivariate	NSP2+VP6	*Clostridium_sensu_stricto*	1.81	dose1_shedding_NSP2VP6	yes	6.624	2.367	56	13	0.00743	0.10697	23.21
week6	dose 1 shedding	multivariate	NSP2+VP6	*Klebsiella*	2.41	dose1_shedding_NSP2VP6	yes	2.670	1.034	56	40	0.01300	0.17329	71.43
week10	dose 2 shedding	univariate	NSP2+VP6	*Veillonellaceae*	0.17	dose2_shedding_NSP2VP6	yes	1.576	0.344	46	6	0.00004	0.00410	13.04
week10	dose 2 shedding	univariate	NSP2+VP6	*Holdemanella*	0.13	dose2_shedding_NSP2VP6	yes	0.118	0.032	46	10	0.00057	0.03075	21.74
week10	dose 2 shedding	univariate	NSP2+VP6	*Chryseobacterium*	0.19	dose2_shedding_NSP2VP6	yes	1.325	0.478	46	8	0.00815	0.07268	17.39
week10	dose 2 shedding	multivariate	NSP2+VP6	*Veillonellaceae*	0.17	dose2_shedding_NSP2VP6	yes	1.726	0.369	46	6	0.00004	0.02250	13.04
week10	dose 2 shedding	multivariate	NSP2+VP6	*Holdemanella*	0.13	dose2_shedding_NSP2VP6	yes	0.108	0.034	46	10	0.00299	0.20955	21.74
week10	dose 2 shedding	multivariate	NSP2+VP6	*Chryseobacterium*	0.19	dose2_shedding_NSP2VP6	yes	1.529	0.505	46	8	0.00444	0.24345	17.39

The statistical R package MaAsLin2 was used for analysis. The default significant association with a maximum Q-value of 0.25 was used.

The MaAsline2 is a two-sided test because MaAsLin2 (Microbiome Multivariable Association with Linear Models) applies generalized linear models (GLMs) or linear mixed models (LMMs) to test for associations between microbial features and metadata variables.

All MaAsLin2 identified taxa associated with vaccine variables and with a minimum abundance of 0.1% and with a minimum prevalence in at least 10% of samples are shown here.

In addition, only taxa with a minimum of 3 samples in either the yes and no vaccine variable response group have been included in this table.

Column header description:

Column header description:

Analysis group = each analysis group was analysed separately

Method: univariate analysis (using vaccine variables only) and multivariate analysis (using vaccine variables together with additional metadata)

Feature: The microbial taxon associated with the metadata

min 0.1% abundance in total cohort = only taxa with a minimum abundance of 0.1% in the total cohort/dataset are shown here, but all taxa were used for MaAsLin2 analysis

Metadata: The variable name (i.e. metadata) associated with the microbial feature.

Value: for categorical features, the specific feature level for which the coefficient and significance of association is reported.

min 3: only vaccine variable data with at least three yes and/or three no responses for the given vaccine variables were included.

Coef: the model coefficient value (effect size). Coefficients for categorical variables indicate the contrast between the category of value and the reference category.

Stderr = the standard error of the model

N = the total number of samples used for analysis in the model

N.not.0 = the total number of these samples in which the character is not zero.

This table only shows taxa that were present in at least five samples.

pval and qval: the nominal significance of this association and the corrected significance calculated with p.adjust using the correction method "BH", respectively.

The last column shows the number of samples in which the taxa were found. Only taxa present in at least 10% of the samples are shown.

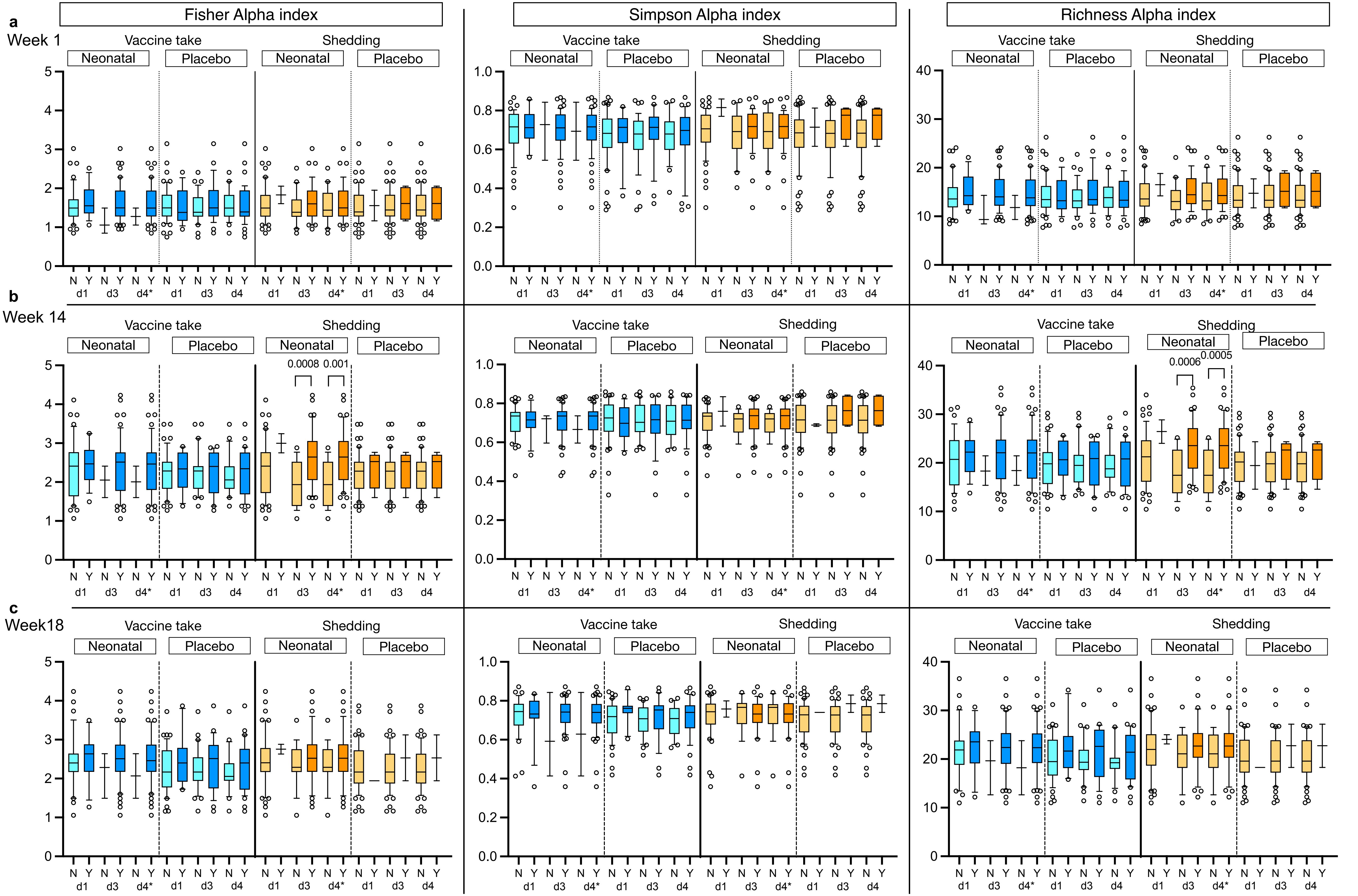

Supplementary Fig. 1 Alpha diversity analysis for the Rv3-BB Indonesia study cohort in participants with positive or negative vaccine response in the Neonatal vaccine schedule group
The alpha diversity was analysed between the negative vaccine response group (N) and the positive vaccine response group (Y) for the vaccine variables "Vaccine take" and "Shedding" at week 1 shown in **a**, at week 14 shown in **b**, and at week 18 shown in **c**. The analysis was conducted on three vaccine doses: dose 1 (d1), dose 3 (d3), and dose 4 (marked as d4*as this time point is after three doses of vaccine and one dose of placebo). The data were tested for normal distribution using the Shapiro-Wilk and Kolmogorov-Smirnov tests. For normally distributed data, a two-tailed unpaired t-test was employed, whereas for non-normally distributed data, a two-tailed unpaired Mann-Whitney test was used. Data are presented in a box and whisker plot. The box extends from the 25th to the 75th percentile and the line in the middle is plotted at the median. The whiskers represent the 10-90 percentiles. All data points outside the 10-90 percentile are shown. All the individual numbers used for box plot generation are presented in Supplementary Table 4. All statistical tests were conducted in GraphPad Prism 10 for macOS (v 10.3.0).

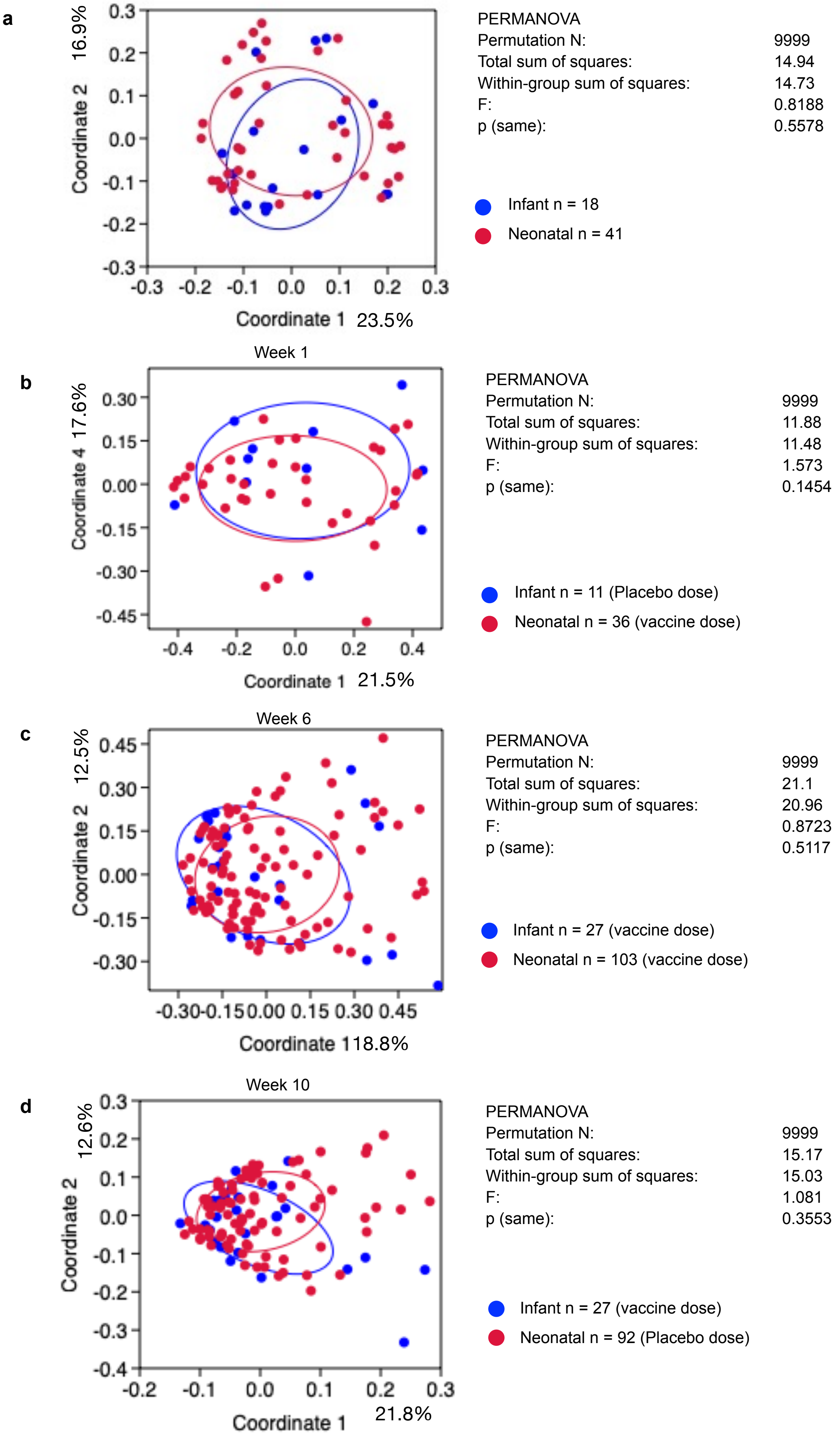

Supplementary Fig. 2: PCoA showing that the microbiome in the RV3-BB Malawi infant and neonatal schedule groups are not different
The PCoA based on the Bray-Curtis distance matrix performed between the infant and neonatal groups in the Malawi RV3-BB vaccine cohort showed that the microbiome was not different between these two groups at **a** baseline, **b** week1, **c** week6, and **d** week 10. The PERMANOVA test for beta diversity confirmed that the microbiome was not statistically different between the infant and neonatal groups.

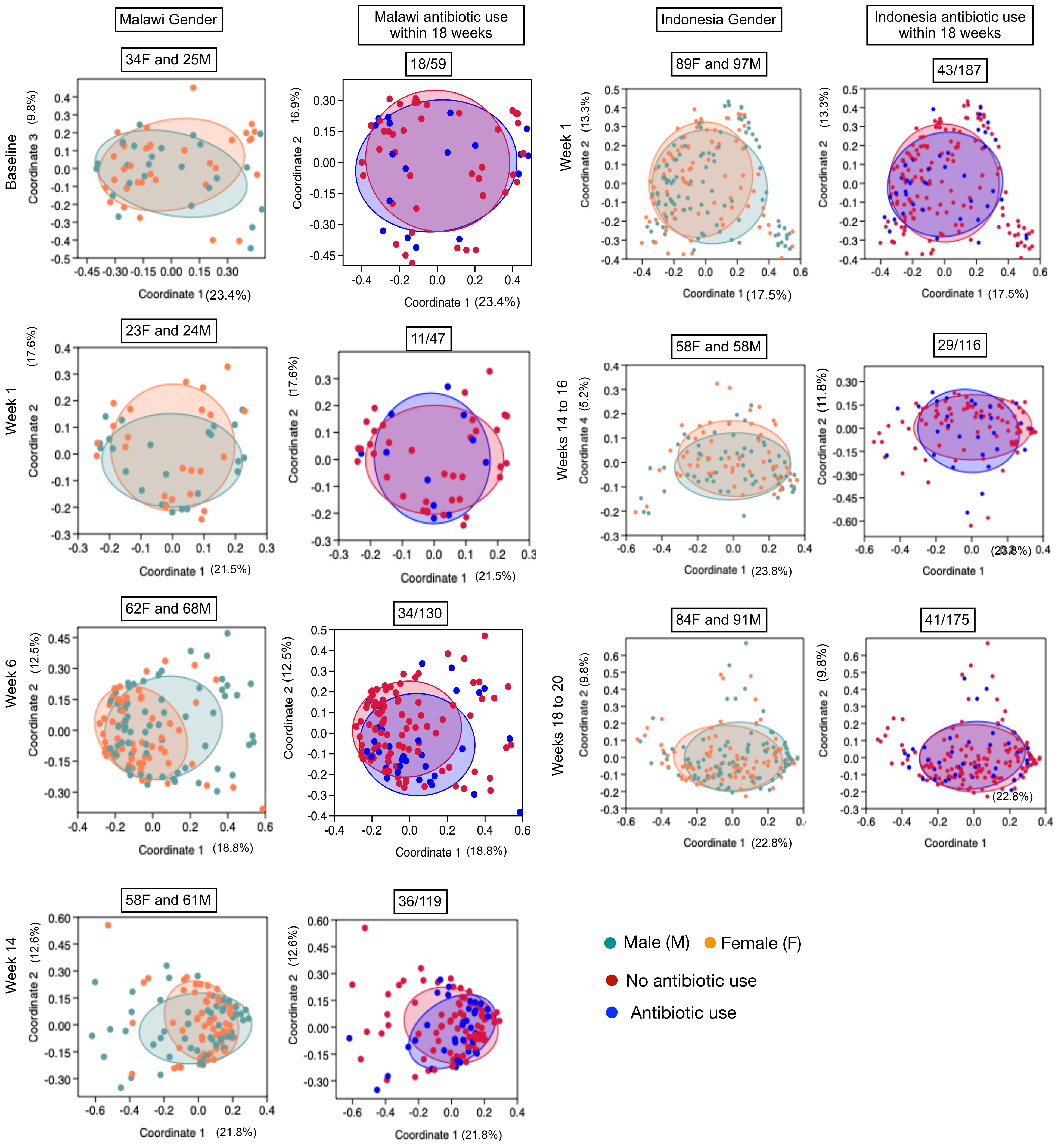

Supplementary Figure 3: PCoA for gender and antibiotic use in the RV3-BB Malawi and Indonesia study cohorts

PCoA based on Bray-Curtis distance matrix between gender and antibiotics free group and the group who has received antibiotics during the entire 18 weeks IP dose period did not reveal different bacterial clusters between the different groups and timepoints analysed.The explained variance for the coordinates are shown in brackets next to the coordinate axis label. PCoA was done with the Paleontological Statistic software package for eduction and data analysis (v PAST 4.04) with 9999 permutations on total sum scaling (TSS) transformed data. The confidence ellipse was drawn at 60%.

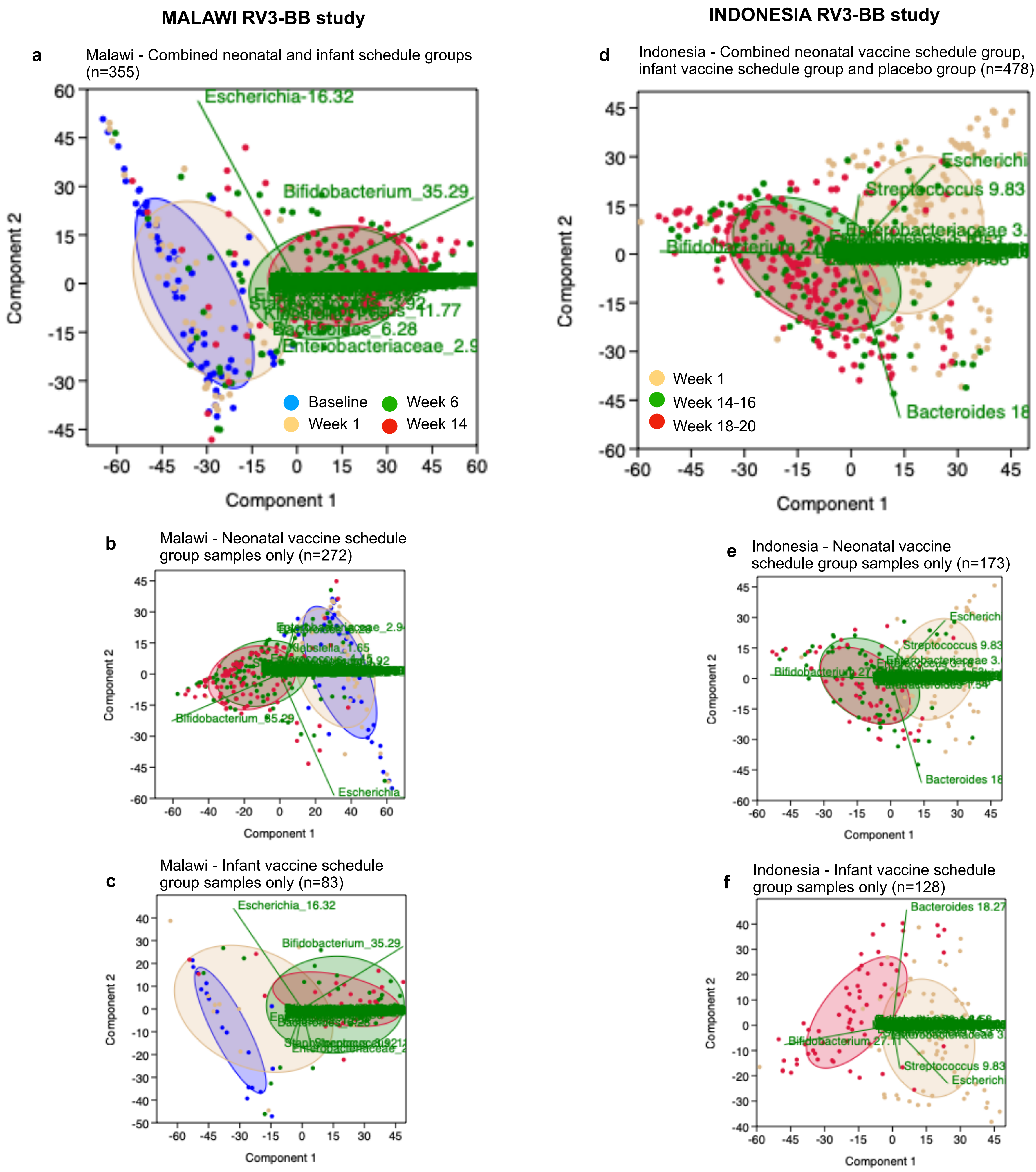

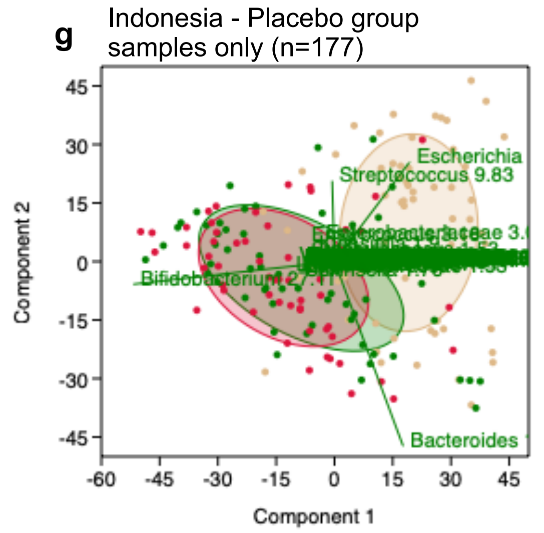

Supplementary Figure 4: Principal Component Analysis (PCA) for timepoints in the RV3-BB Malawi and Indonesia study cohorts demonstrate the existence of age-related distinct clusters

a PCA conducted on the Malawi study all samples combined (n=355), **b** using neonatal vaccine schedule group samples (n=272), **c** and infant vaccine schedule group samples (n=83) shows the presence of two age-related distinctive bacterial clusters (at baseline and week 1 samples; and at week 6 and week 14). This distinct age-related bacterial cluster was also observed in the Indonesia study cohort; **d** in the combined (neonatal vaccine schedule group and infant vaccine schedule group; n= 478), **e** in the neonatal vaccine schedule group at week 1 compared to the week 14 and 18 cluster (e); **f** and in the infant vaccine schedule group the week 1 compare to week 18. **g** In the placebo group this age-related bacterial cluster was also observed between the week 1 and week 14 and 18 (g). The PCA plot was overlayed with a biplot showing the most distinctive bacterial cluster responsible for the separation.
