# Supplementary material for: Early-life gut microbiome associates with positive vaccine take and shedding in neonatal schedule of the human neonatal rotavirus vaccine RV3-BB
Source: Nat Commun. 2025 Apr 11;16:3432. doi: 10.1038/s41467-025-58632-6 (PMC11986061; doi:10.1038/s41467-025-58632-6)
Supplement: Supplementary file 2 — Description of Additional Supplementary Files [file 41467_2025_58632_MOESM2_ESM.pdf]

Early-life gut microbiome is associates with positive vaccine take and shedding in neonatal schedule of the human neonatal rotavirus vaccine, RV3-BB

Josef Wagner[1,2,3]*, Amanda Handley[1,4], Celeste M. Donato[1,3], Eleanor A. Lyons[1], Daniel Pavlic[1], Darren Suryawijaya Ong[1], Rhian Bonnici[1], Nada Bogdanovic-Sakran[1], Edward P.K. Parker[5], Christina Bronowski[6], Jarir At Thobari[7,8], Cahya Dewi Satria[8], Desiree Witte[6,10], Hera Nirwati[9], Khuzwayo C. Jere[6,10], Ashley Mpakiza[10,11], Emma Watts[1], Ann Turner[10], Karen Boniface[1], Jonathan Mandolo[10], Frances Justice[1], Naor Bar-Zeev[6], Miren Iturriza-Gomara [6,12], Jim P. Buttery[1,3,13], Nigel A. Cunliffe[6], Yati Soenarto[8], Julie E. Bines[1,3,14]*

Legend of Supplementary Data files

Supplementary Data 1

Description: Absolute and relative abundances of bacterial taxa in the RV3-BB Malawi and Indonesia cohorts and in the Rotarix Malawi and India cohorts

Supplementary Data 2

Description: Multivariable statistical framework analysis (MaAsLin2) for finding associations between microbial taxa and vaccine variables in the Malawi and Indonesia RV3-BB cohorts

Supplementary Data 3

Description: Summary of published studies reporting microbiome analysis in relationship to rotavirus vaccines compared to this RV3-BB study in Malawi and Indonesia
